# Large adrenal tumor in a 76‐year‐old man

**DOI:** 10.1002/ccr3.3749

**Published:** 2021-01-08

**Authors:** Andreas Kiriakopoulos

**Affiliations:** ^1^ Department of Surgery 5th Surgical Clinic “Evgenidion Hospital” National and Kapodistrian University of Athens Medical School Athens Greece

**Keywords:** adrenal metastasis, adrenal tumor, lung adenocarcinoma, PET/CT, retroperitoneal tumor

## Abstract

Among various adrenal tumors, metastatic ones are the most common. PET/CT scanning facilitates early detection. Occurrence of isolated and synchronous metastasis is very rare and poses serious diagnostic and therapeutic challenges.

## Graphical abstract

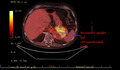

## 1 CASE PRESENTATION

A 76‐year‐old man was found with a 12.3 cm, hormonally inactive, left adrenal mass by abdominal CT. FDG PET/CT scan showed a malignant adrenal tumor (SUV_MAX_ uptake:18) and revealed a 1.1 cm nodule at the upper right lung field (SUV_MAX_:5,9). Left adrenal resection confirmed the adrenal metastasis.

**FIGURE 1:**
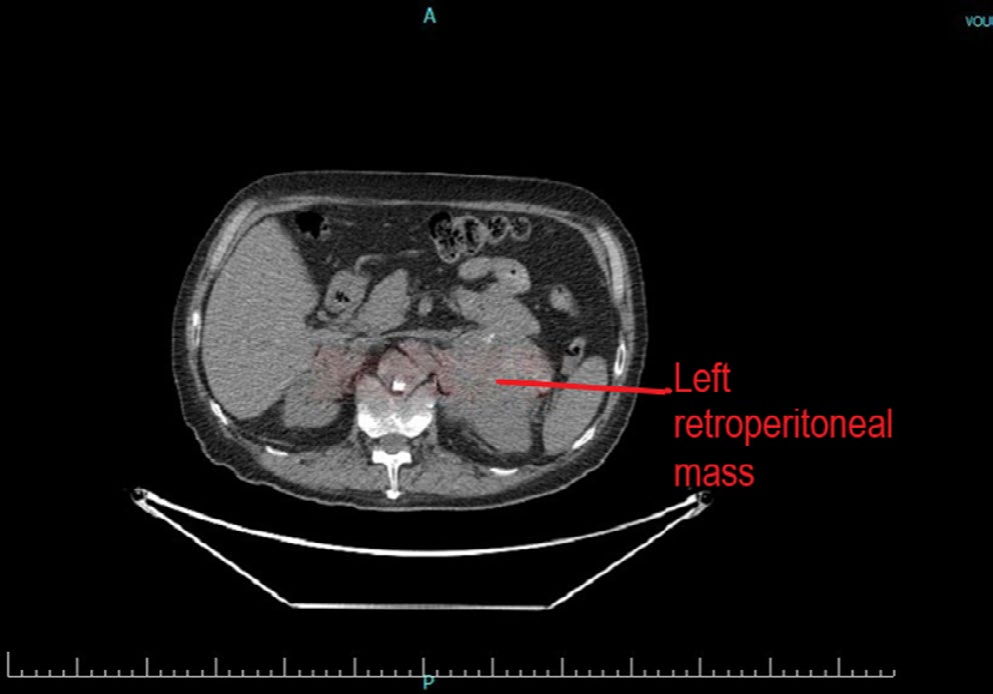
Left adrenal inhomogeneous mass with irregular borders

**FIGURE 2:**
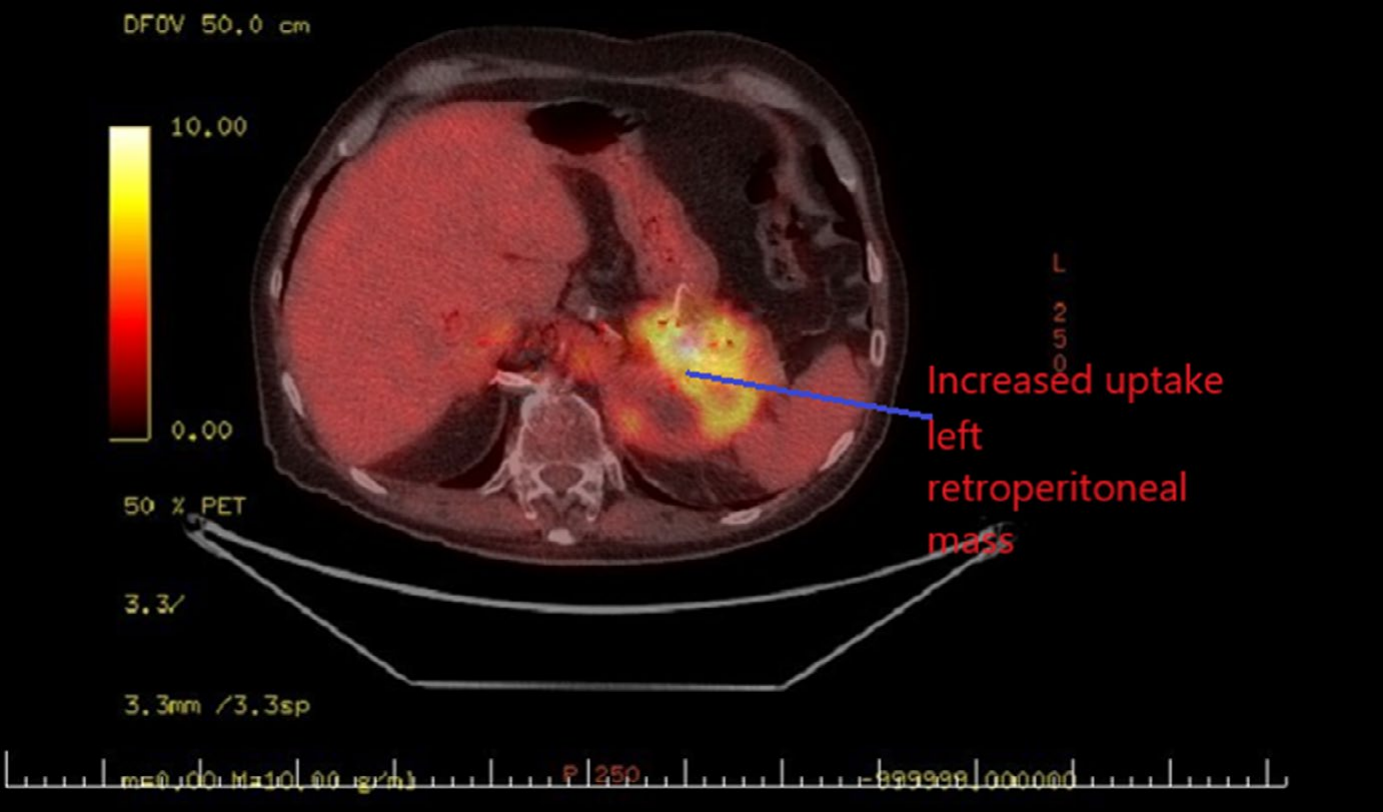
PET/CT scan showing a left adrenal tumor with SUV_MAX_ uptake:18

**FIGURE 3:**
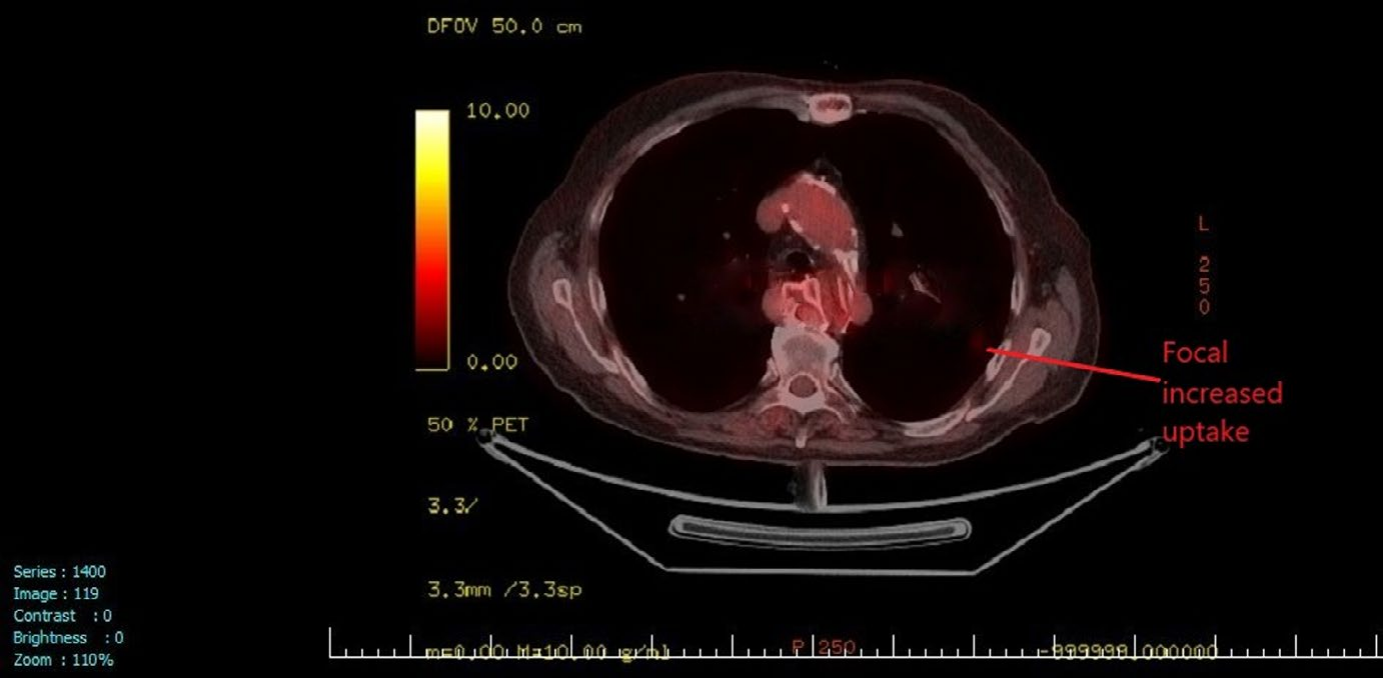
PET/CT scan revealing a 1.1 cm nodule at the upper right lung field (SUV_MAX_:5,9)

A 76‐year‐old man was found with a left adrenal mass after complaints of weight loss and anorexia during the past 2 months. His past medical history included heavy cigarette smoking and signs/symptoms of generalized vascular disease. Abdominal CT revealed a 12.3 cm inhomogeneous mass with irregular borders (Figure 1). Endocrine evaluation showed normal plasma metanephrines, normal 24‐hour urinary cortisol, and normal 17‐OH progesterone, androstenedione, and DHEA‐S levels. Fused FDG PET/CT scan (Figure 2) confirmed the presence of a malignant adrenal tumor with a SUV_MAX_ uptake: 18 and revealed an increased SUV_MAX_: 5,9 of a 1.1 cm nodule at the upper right lung field (Figure 3).

### 1.1 Q: Which is the primary tumor?

Left adrenal resection confirmed the presence of adrenal metastatic adenocarcinoma from the lung primary. The adrenal gland is a potential site of metastasis from various malignancies. Adrenal metastases are the second most common type of adrenal masses after benign adenomas, and most of them are seen in patients with disseminated cancer and rarely are isolated. Timewise, metastases can be synchronous or metachronous.^1^ Differentiation between a metastatic tumor from a primary adrenal mass can be challenging and requires the selective use of radiologic imaging studies, serologic tests, and, in highly selected cases, open adrenal resection.^2^

## CONFLICT OF INTEREST

None declared.

## AUTHOR CONTRIBUTION

Andreas Kiriakopoulos MD—author

## ETHICAL APPROVAL

There are no identification details of patient, and an informed consent was taken.

## Data Availability

All images and information regarding the case are available upon reasonable request.
